# A Practical Treatise on Inflammation, Ulceration and Induration of the Neck of the Uterus, with Remarks on the Value of Leucorrhea and Prolapsus, as Symptoms of Uterine Diseases

**Published:** 1847-03

**Authors:** 


ART. II.—*A Practical Treatise on Inflammation, Ulceration and Induration of the Neck of the Uterus, with Remarks on the Value of Leucorrhea and Prolapsus, as Symptoms of Uterine Diseases.* By JAMES HENRY BENNET, M. D., Licentiate of the Royal College of Physicians: formerly House Physician (by concours) to Hospitals St. Louis, Notre Dame de la Pitie, &c., Paris, &c. &c. Philadelphia: Lea & Blanchard. 1847. 12mo., pp. 165.

The substance of this work originally appeared in the London Lancet, in a series of papers, which attracted considerable notice, and were pretty extensively copied by medical periodicals.—The author has been induced, by the interest manifested toward those communications, to re-produce them, with some additions, in a bibliographical form.

Dr. Bennet appears to have enjoyed large opportunities for cultivating the study of uterine affections at the various Parisian Hospitals, having for three years visited them as a pupil, and for four years held the position of a resident medical functionary. No city in the world can offer such a field of observation, in this province of pathology, as Paris. There Hospitals exist not only for sexual diseases exclusively, but for their various forms specially. The wise and liberal policy of making all eleemosynary institutions subservient, in the fullest degree, to medical knowledge, is there adopted, pupils from all countries being freely admitted to their advantages, and even eligible alike to their medical offices. In addition to these general regulations, the system of licencing females prostitutes, and subjecting them to weekly examinations, whatever may be thought of its moral aspect and consequences, while it affords a check upon the dissemination of disease, offers unusual opportunities for scientific observation; and these opportunities are sttll further increased by the use of the speculum, which is the rule, instead of the exception, at Paris, the reverse being the case elsewhere. With all these advantages, it is not strange that the diseases of the female sexual system are much better understood in France than by the pathologists of other countries—and that improved views of both the patholgy and treatment of these diseases should emanate chiefly from that quarter.

Dr. Bennet's work is to be regarded as mainly an exposition of French theory and practice as regards the subjects of which it treats. He claims to have advanced ssme original practical observations, aud we do not deny that it is so. But it is manifest to the reader, that the merit of his labours consists, chiefly, in rendering available to the English reader, the doctrines and methods entertained and practiced by the distinguished surgeons of the hospitals where he pursued his observations. He states, however, and we do not, of course, doubt the fact, that his own experience in Private practice for several years since his return from Paris, has confirmed the correctness of the practical principles imbibed during his connections with the hospitals of that city. That these principles were in a great measure novel to English Practitioners, although by no means so at Paris, we can readily believe, notwithstanding the proximity of the two countries. The jealousy felt by the English toward their Gallic neighbors, doubtless interupts to a considerable extent, the ready transmission of improvements across the English channel. Scientifically speaking, the profession of this country stand in nearer relation to France than that of Britain. This fact is illustrated in the present instance; for we believe we are correct in the assertion, that the greater portion of Dr. Bennet's work was already familiar to American Physicians, as the views of prominent Parisian practitioners who have devoted particular attention to this specialty. Still these views are not so generally and fully known with us as they deserve to be, and we think, therefore, that we shall render an useful service, at least to a portion of our readers by presenting the more important matter contained in the work referred to.

It seems to be well ascertained, not only that the *Cervix* is by far more frequently affected with disease than any other portion of the uterus, but, that in a very large proportion of cases which come under the denomination of Leucorrhea, this part is chiefly involved. This fact is susceptible of explanation from its anatomical structure being cellular, and more vascular than any other portion of the uterus; from being exposed to friction upon the parietes of the vagina, and to contusion in sexual intercourse ; and from its liability to injury in labor. Dr. Bennet attaches considerable importance to marriage and the bearing of children in producing a liability to disease of the cervix. The condition of this part in those who have conceived is so modified as to occasion greatly increased aptitude to disease, and leucorrhea under these circumstances is almost invariably associated with lesions of the uterine neck. In virgin females, opportunities for observation being necessarily limited, the frequency of inflammation, ulceration, &c, of the cervix is not well ascertained. From the speedy relief which the usual remedies afford in blennorrhagic discharges occurring in that class of patients, it is presumable that this complication either does not exist, or exists in a slight degree only. Diseases of the cervix, therefore, may be practically considered, as for the most part peculiar to married females, or those exposed to sexual intercourse, and to those who have borne children; and between these two classes of patients the differences in the susceptibilty of that part to disease, and in its severity and rebellious character, are such that Dr. B. treats of each separately, under distinct heads.

*Chapt.* 1. *Inflammation, ulceration, and induration of the cervix uteri in women who have not borne children.*

The affection is generally limited to the mucus membrane; that degree of engorgement and induration which characterises chronic cases in those who have borne children, seldom being present. Leucorrhea is usually present, but sometimes is so slight as to be scarcely perceptible. Pain in the loins is usually complained of, and sometimes in the hypogastric region deeply located. Intercourse is painful. A feeling of heat in the vagina is sometimes present.—The sensations communicated by applying the finger to the cervix under these circumstances, are described as follows:

*Toucher.*—On examining by the toucher, the neck of the uterus is found hotter than the lower part of the vagina; it has lost its unctuous, greasy feel; its volume is more or less increased, as also its elasticity, owing to its being more or less congested. Still there is no general or deep-seated induration of its tissue. The surface, likewise, is smooth and unresisting, unless ulceration has set in. When this is the case, it is at the orifice of the uterine cavity that the ulceration commonly begins, and from that region that it spreads; owing, no doubt, to the greater tenuity and delicacy of the mucous membrane. Pathologists generally state that the ulceration may be recognised, by its producing the sensation of a velvety surface when the finger is passed lightly over it.—Finding, however, that this peculiar sensation is so difficult to appreciate in this form of the disease, that those who rely upon it alone must be as often wrong as right, I have endeavored to discover a more correct guide, and have ascertained that ulceration of the mucous surface, however limited, almost invariably gives rise to slight induration of the tissue underneath, which induration is very perceptible to the touch. In the form of ulceration that we are now examining, the induration to which I allude is quite superficial, not extending to the central tissue of the uterine neck. It is merely a thickening of the ulcerated mucous membrane, and of the sub-cellular tissue, most perceptible at the circumference of the ulceration; yet it is easily appreciated by the finger of one who is accustomed to look for it, and to him is a valuable symptom. The superficial induration is generally felt most distinctly at the edge of the uterine lips, where the mucous membrane passes into the cavity of the neck, and where, consequently, two mucous thicknesses are approximated by the folding of the membrane. Although I have found this symptom of great assistance in the diagnosis of ulcerations, I must confess, nevertheless, that it is not infallible. In the very first stage of ulceration, induration may not yet exist, whilst, on the other hand, the ulceration may heal, and the superficial induration may remain for a few days. When the inflammatory induration extends to the entire substance of the cervix, as it generally does if the ulceration exists in women who have had children, the superficial induration is necessarily lost in the general hardness. Pressure on the inflamed and ulcerated cervix will often, not always, occasion slight pain, which is never the case in the healthy state.

We enter our protest against the pseudo-refinement of the expression "*toucher*." Why not say examining by the *touch*, or with the finger? The modern substitute is a solecism in language, for the *toucher* is not the *touch*, nor the *finger*, but the individual who examines.

The sensations derived by touching, in Dr. B.'s estimation, are far less definite than those obtained by employing the vision by means of the speculum. We quote the Author's description of the visible appearances:

*Speculum.*—On examination by the speculum, a certain quantity of mucoso-purulent matter is always found at the superior region of the vagina, even when the lining membrane of that organ is not inflamed; the cervix uteri is generally increased in size, but seldom so much as not to be admitted into the cavity of an ordinary sixed conical speculum, the one I generally use, and by far the most convenient and the least painful to the patient. The tumefaction is mostly greatest on the upper lip, which is the largest one of the two in the healthy condition; it is therefore often necessary, in order to expose the orifice of the os, to raise the speculum towards the pubis, and by thus slightly pressing with the superior edge of the instrument on the anterior lip, to push it back, and allow the inferior os to enter its cavity. Even if the cervix uteri is too large to be admitted at once into the speculum, by thus alternately depressing its different parts, the entire organ may successively be brought fairly into view. When inflamed, the tumefied cervix presents a more or less intense red, glistening hue, instead of the pale dull whitish color, which is natural to it. On its surface may frequently be seen small white or red vesicular, or papular, elevations, the result of distention of the mucous follicles, or of their hypertrophy. Different forms of inflammation have been admitted by some writers, founded on this appearance, but without any practical utility whatever. When the mucous membrane is ulcerated, the glossy appearance of the membranous surface is lost, and a number of vascular granulations, of a vivid red hue, are seen covering the ulcerated region, after the mucus has been wiped away with a pledget of lint—a necessary precaution. Sometimes the ulcerated surface appears raised above the adjacent level, whilst occasionally, on the contrary, it appears depressed, When the ulceration is at the entrance of the os uteri it is often difficult to discover, unless the uterine lips slightly separated. There is generally a mass of semi-transparent mucus occupying the cavity of the os uteri. The ulceration may be so superficial and slight as to be scarcely perceptible, or extend over a considerable portion of the cervix. In many cases, the pressure of the edge of the speculum, or even of the pledget with which the mucus is wiped off, occasions a slight oozing of blood from the abraded or ulcerated surface. This also frequently occurs when patients thus affected expose themselves to intercourse—a fact of which they themselves are often cognizant. Menstruation is generally more painful than in the healthy state, owing to the temporary congestion of the uterus increasing the inflammatory irritation of the cervix. Indeed the occurrence of the various symptoms of painful and difficult menstruation, when coupled with a leucorrhœal discharge, may be considered, in most cases, as pathognomic of inflammation and ulceration of the cervix. Occasionally slight irritation of the urinary organs is present, giving rise to frequent desire to urinate. The anoyance and distress of mind which the local symptoms sometimes produce, coupled with the leucorrhœal discharge, when it is abundant, may react more or less on the general health, and give rise to dyspepsia, palpitation, general weakness, &c.

We have, on a former occasion taken occasion to make some remarks on the use of the Speculum in private practice. There can be no question that diseases of the cervix would be much more satisfactorily treated, if the practitioner, in all cases, prepared himself by a knowledge of the actual condition of the parts from ocular inspection. We would think it absurd to prescribe for pharyngitis without an examination of the throat, yet these are no reasons, except those of delicacy, why we should be better satisfied to treat the affections under consideration without the advantage of knowing the actual appearances of the parts. It is doubtless the case that if these affections are properly recognised, and efficiently treated, they are, for the most part, promptly cured. As it is, they are among the *opprobria* of the profession. When we consider what a host of secondary ailments follow in their train, and how many females suffer for years from their consequences, life, in fact, often being rendered miserable to themselves and useless to others, if, indeed its duration be not shortened, the subject certainly appears one of vast importance. Taking a scientific and humane view of the matter, there can be no doubt that the speculum, instead of being rarely used, as it now is, at least in so far as our knowledge extends, should be considered quite as essential to the diagnosis of the diseases of the female sexual organs, as the stethoscope in affections of the chest. There are very few females who refuse their consent to its use in cases where its importance is fully and properly stated; let the necessity for its application in nearly all cases be in like manner represented, and generally understood, and the practitioner will soon find it much more difficult to avoid availing himself of it, than he now does in convincing, occasionally, patients of its necessity. We are satisfied that all that is needed, is for the profession generally to appreciate its utility, and unite in the habit of resorting to it. We must confess that we have been recreant to our duty in this particular. We shall try to do better hereafter, and we hope that the more common use of the instrument by practitioners, will render it easier for ourselves and others to carry this good resolution into practical operation.

We shall speak here of the treatment of the forms of disease which have described, deviating somewhat from the collocation of subjects which Dr. Bennet pursues. He defers the subject of treatment until he has considered the affections in those who have borne children. We quote his words:

The remedies usually resorted to in general vaginitis are here indicated. The most efficacious is, undoubtedly, the solid nitrate of silver, lightly drawn over the inflamed cervix, and over that part of the inflamed parietes of the vagina which generally participates in the inflammation. The peculiar influence which the nitrate of silver exercises over inflammation of mucous membrane, is recognised by nearly all practical medical men, and its use, either in solution, as an ointment, or solid, is daily becoming more general. On the cervix and on the vagina, it exercises the same beneficial effect as on the eyes, the mouth, the urethra, &c., and proves a most valuable remedial agent.

After the mucous surface has thus been slightly cauterized, emollient or slightly astringent injections (acetate of lead, or sulphate of zinc) must be used three or four times a day; complete rest must be enjoined; sexual congress strictly prohibited; and the general health attended to. The disease is nearly always so entirely a local one, that it is not necessary to resort to any general medication, unless the state of the health seems to demand it. A brisk purgative or two, however, can do no harm, and will often do good.

On the parts being examined in the course of a week or ten days, the inflammation will generally be found to have subsided, unless it co-exist with blennorrhagia, in which case it will be much more difficult to eradicate, and requires the same treatment as in the other regions of the sexual organs. Sometimes, nevertheless, it will be found advisable or necessary to re-apply the nitrate of silver to the mucous surface a second or a third time. This occurs more especially when the injections have been improperly performed, as is usually the case if the patient is not instructed as to the way in which they should be used. Females generally use vaginal injections in a stooping posture, and the result is, that the injected fluid falls out of the vagina, without having attained the uterine neck. This M. Ricord has proved to demonstration by the following experiment:—With the assistance of the speculum he introduced a piece of lint into the upper part of the vagina, and then, carefully withdrawing the instrument, told his patient to use the injection syringe in the usual stooping position. On afterwards extracting the lint, he found that it had not even been moistened.

To obtain the full effect of the injection, the patient should recline on the side of a bed, or of a lounging chair, elevating the pelvis, so as for the vagina to form an inclined plane, of which the cervix is the most dependent point. The vagina thus retains the injected fluid, like a vase; it penetrates gradually into every part, and remaining in contact with the inflamed tissues for a few minutes, exercises a decided influence on them.

The author admits that it is practible to treat successfully non-ulcerated inflammation of the cervix, and slight ulcerations, by injections, and other remedies, without ascertaining the disease accurately with the speculum, and making the local applications directly in the manner just indicated. He does not, however, in this concede enough; for we have ourselves in a multitude of cases of long continued leucorrhea, presenting symptoms characterising, not only inflammation and slight ulceration, but engorgement and induration with deeper ulcerations, by means of injections conjoined with general remedies &c. The reason why the ordinary treatment is so generally unsuccessful is, that in nine cases out of ten, injections are so slovenly administered as to be of no service whatever; as stated by Ricord in the foregoing extract, the fluid frequently never reaches the os tincæ. We have of late years taken especial pains to give to our patients minute instructions concerning the use of these means, committing the duty to a faithful nurse whenever practicable, and the difference in the results has been striking. Important, however, as this point is, we do not mention it as superseding in any degree the advantage of the more accurate application of remedies with the aid of the speculum.

The foregoing treatment applies only to inflammation unaccompanied by ulceration or induration. When ulceration is present, cauterization of the ulcerated surface is the remedy. Dr. B. states that "if the inflammation has not penetrated to the deep tissues of the cervix, and there is no general inflammatory hypertrophy of the organ, *superficial* cauterization, combined with emollient and astringent injections, rest of the organ and of the system and attention to the general health, are all that is required, and, will generally effect a cure in from four to six or eight weeks."

Several agents are used for cauterization. The chief are, the nitrate of silver, the acid nitrate of mercury, actual cautery, and the hydrate of potassa, or the potential cautery. The author confines himself to nitrate of silver, the acid nitrate of mercury, and the caustic potassa, for purposes of superficial cauterization.— The use of the actual cautery is peculiar to M. Jobert, (De Lamballe) Surgeon to the Hospital St. Louis. The nitrate of silver, which, so far as we know, is exclusively used in this country, probably answers every object, and is more easily and safely employed that the others. The following are the directions for its use which are laid down by Dr. B.:

When recourse is had to the nitrate of silver, its application may be repeated every fourth or fifth day, whereas the acid nitrate of mercury should not be applied oftener than once a week. The eschar formed by the former, being much more superficial than that formed by the latter, falls much sooner. In either case, whenever cauterization is restored to, whatever be the agent, the blood or mucus which covers the cervix must be wiped off before the caustic is applied, otherwise the greater part of its power is lost in coagulating these fluids. This can be done conveniently by means of a little lint or sponge, held on a pair of long forceps. Those which I use are thin, about ten inches in length, and made on purpose. The solid nitrate of silver, fixed in a long porte-caustic, must be drawn two, three, or four times over the ulcerated surface, according to the effect wished to be obtained.

In order to apply a fluid caustic, the following plan should be resorted to :—A small thin stick, about a foot in length, having been chosen, is formed into a brush, by inserting between its divided extremities a little wool, lint, or old linen, which is then fastened by a few turns of thread. These little brushes may be made *extempore*, and being of no value, can be thrown away when they have been used. The brush, having been introduced into the acid, should be pressed against the rim of the bottle, in order that it may be merely moistened with the caustic, and then drawn over the ulcerated surface. A little water must then be injected into the speculum before withdrawing it, in order to absorb any super-abundant acid. This precaution is not absolutely necessary, if care-has been taken not to use too moist a brush. Owing to the powerful cauterizing properties of acids, it is perhaps, as well, however, for a person unaccustomed to the treatment of these diseases, to adopt the precaution. In this case a syringe, holding about half-a-pint of water, should be used, and the water injected before the speculum is withdrawn. By changing the position of the pelvis, the fluid may afterwards be made to fall into the basin. I often merely pour a little olive oil into the speculum ; the effect obtained is the same.— When the nitrate of silver is employed, no precaution is necessary. The contact of the uncombined caustic with the neighboring parts, nearly always inflamed, can only be productive of benefit. In cauerizing the cervix, the speculum must be *firmly* applied to the parts, so as to protect the vagina from the action of the caustic.

The application even of the most energetic caustics, such as the actual and potential cautery, to the cervix uteri, gives little or no, pain. This is owing to the absence of spinal nerves in that part. It is unattended with danger of metritis, or peritonitis, and only gives rise occasionally to hysterical symptoms. It is not, however, devoid of evils, if improperly or carelessly made. If the caustic substances are allowed to come in contact with the vagina, which is endowed with vivid sensibility, excessive pain would be produced, and inflammation, with its consequences, might be the result.

Emollient injections should be employed after the cauterization, such as milk and water, infusion of linseed, decoction of marsh, mallow, &c. If the inflammation embraces the parietes of the vagina the injections should be of an astringent character, Solutions of the sulphate or acetate of zinc, of the sulphate of alumina and potassa, &c., may be employed under these circumstances.

Quietude, sometimes rest in a horizontal posture, abstinence from sexual intercourse, are important to ensure the cure. Tonics, and the various preparations of iron, are beneficial. The astringent injections are to be suspended, of course, during the menstrual periods.

Before leaving this portion of the subject, we will allude to the views of Velpeau, which we have met with in a clinical report communicated by an American physician now at Paris (Dr. F. Willis Fisher,) for the Boston Med. and Surg. Journal, Velpeau regards nearly all the non-malignant lesions of the uterine neck, called by Dr. Bennet, and others, ulcerations, as granulations. He says "one seldom finds other lesions than granulations of the neck among those generally called ulcers." He is disposed to place more reliance on the touch than the use of the speculum, although he by no means advocates the omission of the latter. Even if the touch should suffice in all cases for diagnosis, the speculum is necessary in order to make the cauterizing applications. We will quote from the report referred to, some of the remarks of Velpeau upon the affection under consideration. He says:

"The granulations of the neck are almost always indicated by a white discharge more or less abundant. To this sign may be added pains in the stomach, the patient complains of growing thin, and of suffering in the kidneys and groin. When a patient presenting these symptoms is touched by the finger well extended, the neck is found slightly augmented in size; it presents the sensation of some kind of grain, or like a raspberry but is firmer. These characters are easy to recognize after one has acquired some skill. The granulations are more easily recognized by the touch than by the speculum. In using the speculum, one is certainly more exposed to error unlest he has great skill. This may appear singular, yet it is true. When the instrument is introduced, the mucous membrane of the vagina presents two folds which may be mistaken for the neck; or, at other times, only one side of the neck may be visible, which may be healthy whilst the other is diseased. The finger is not liable to these mistakes; it is the best method, but does not prevent the use of the speculum. These two methods united give a degree of certainty not acquired by either separately. The comparison of the sensation furnished by the granulations to a raspberry or strawberry, is sufficiently exact. The granulations are found either scattered or in patches, and most frequently occupy the mouth, but sometimes are met with in the interior of the neck. It is in this last case that the speculum is most useful. When the instrument is introduced and the neck embraced by its extremity, by pressing a little it enters the open mouth, and if there is a muco-purulent discharge discovered, granulations, without doubt, exist in the interior. These granulations, when observed in the interior of the cavity or upon the mouth, affect only the mucous membrane, the epithelium is destroyed, the papillary layer is exposed, but the tissue of the organ remains healthy. Granulations are rarely complicated with marked engorgement. This disease, so frequent at the present time, presents no other varieties; sometimes, it is true there happen excoriations which terminate in ulcerations, but these do not merit attention and the disease is the same. The question of the most importance is this, are granulations dangerous? Some practitioners assert the affirmative; they believe them to be the cause of cancer in the womb; but this is not proved. There are so many women who have these granulations and who keep them a long time without having cancer, that is doubtful that this last disease, so difficult to cure, can be a consequence of the first. This granular state of the neck can be cured in almost every case. We do not hesitate to say, that nineteen of twenty cases are cured, and generally with the same treatment. Cauterization of the granulated parts is the remedy par excellence. No person is ignorant of it, every one practices it, but not in the same manner, nor with the same caustic. One can cure them probably with any caustic, but not so easily nor so quickly, each caustic possessing a specific virtue of its own. For granulations, the nitrate of mercury is the most efficacious, and the most convenient, and is never injurious. The manner of procedure is very simple: a small pledget of lint moderately wet with liquid, is passed over the granulated parts, and is forced into the neck if granulations exist in the interior. These cauterizations are repeated every eight days during five or six weeks; they need not be continued longer. This precept is neccessary in order to avoid an error injurious to the patient and into which one will certainly fall unless he conforms to it. When the cauterization has been well performed, there remains after it an ulceration, and if the surgeon continues to cauterize whilst it exists, one would never cease, because the ulceration is caused by the cauterization itself. A few baths and tonics complete the treatment, the success of which is nearly certain.

We come now to the second division of the subject, viz.: *inflammation, ulceration, &c., in women who are pregnant, or who have borne children.* We have already alluded to the increased aptitude of the cervix to disease, incident to the modifications produced in it by the puerperal state. Not only does this state induce this greater proneness to disease, but the deeper tissues of the part are much more likely to be affected. Indeed, hypertrophy and induration of the neck, associated with ulcerations, are almost exclusively confined to women who have passed through this state.

It has been a question, whether, in these cases, the ulcerations precede the hypertrophy and induration, or *vice versa.* Some Parisian surgeons have contended that the ulcerations are effects, not causes, of the associated lesions. Dr. Bennet, however, takes a different view, and states that, in some cases, he has been able to ascertain the existence of ulcerations as the primitive affection, and to trace the subsequent enlargement and condensation.

The latter lesions are generally confined to the cervix; but, in some cases, they extend to the body of the uterus. At first, the evidences of acute inflammation are present, viz.: increased heat of the organ, vivid redness, and pain on pressure. These symptoms, after a time, subside, and the case becomes one of chronic hypertrophy. The size of the enlarged neck varies from that of a walnut to that of an egg. Prolapsus is an almost constant attendant upon hypertrophy. The body of the uterus is apt also to be tilted forward, constituting anteversion of that organ, and retroversion of the cervix. The degree to which the body of the uterus descends will depend on the laxity of the vagina. If the structures of this canal fail in affording due support, the uterus may be weighed down by the increased neck to the orifice of the vulva,

The following is Dr. B's. description of the symptoms appertaining to the form of the disease under present consideration:

The condition of the patient is considerably modified by engorgement of the cervix, and the gravity of the disease much increased. The sensation of weight, and heaviness in the hypogastric region, scarcely perceptible as long as the cervix is merely congested, becomes very marked and distressing, especially in walking and standing. Indeed, if the inflammatory hypertrophy is considerable, the patients not unfrequently complain, that whenever they are on their legs they feel as if the womb were on the point of falling out of the pelvis. The deep seated hypogastric pains are increased, and sometimes pressure above the pubes is slghtly painful. The pain in the loins and in the lumbar region is generally continued, and most distressing. Severe pains are also often experienced in the thighs, along the course of the sciatic, obturator, and crural nerves.—these pains, are no doubt, in a great measure, to be attributed to the pressure, aud to the traction exercised by the engorged and prolapsed cervix of the nerves supplied to the uterus by the lumbosacral plexus. They are much more severe when the cervix is ulcerated and engorged, than when it is merely ulcerated. When there is retroversion of the neck, the hypertrophic cervix pressing on the rectum renders evacuation of the fæces difficult and painful. The body of the organ being also thrown forward, may irritate the bladder, and occasion frequent desire to urinate. The presence of the ulcerated and indurated cervix in the cavity of the vagina secreting an abundant muco-purulent fluid, which partly stagnates in the organ, is inevitably followed by the inflamation of its mucous membrane, and by the general vaginitis, which increases the amount of the leucorrheal discharge.

When a patient is in this state, it is not long before the general health begins to fail. Racked with pain, suffering from an abundant leucorrheal discharge, it is impossible that the economy should not suffer. In nearly all cases, the appetite flags, the tongue becomes loaded, the bowels irregular, and in the more severe ones, the patient looses flesh and strength, suffers from continued headache, from want of sleep, and becomes dyspeptic, hysterical, hypochondriacal. As the disease gains ground, when proper measures are not adopted to arrest its progress, all these symptoms increase in intensity ; the patient is nearly unable to leave her bed, and the skin assumes the yellow cadaverous hue, which is occasionally seen in severe chronic inflammatory disease of the uterine organs, and which may be mistaken, and no doubt occasionally is for a symptom of cancerous cachexia. In these severe cases, the inflammation and induration are seldom, if ever, confined to the cervix uteri. They extend more or less to the body of the uterus, giving rise to a subacute form of metritis, indicated by the increased size of the organ, and by the increased severity of the uterine pains. There is also, generally speaking, more or less febrile reaction, especially in the latter part of the day.

Whenever there is even superficial inflamation of the cervix uteri, menstruration is modified by its existence. Indeed, in slight cases, the modification may often materially assist the diagnosis.—The monthly congestion of the uterus generally appears to exacerbate the local inflammation, which, on its side, renders the due performance of the menstrual excretion difficult, probably by abnormally increasing the uterine congestion ; thence intestine uterine pains, increased pain in the loins, and not unfrequently hysterical symptoms. This exacerbation often begins two or three days before the appearance of the menstrua, and lasts for one, two, or more days afterwards. Generally speaking, the ordinary duration of the menstruation is curtailed, and the amount of the excretion diminished ; but it is not always so, for sometimes, more especially in severe cases, flooding will occur at each menstrual epoch, lasting many days. There is but little doubt that the monthly congestion, instead of favoring the resolution of uterine inflammation, as some authors have pretended, is one of the chief causes of its being difficult to overcome.

The sensible changes as determined by the touch and speculum are thus described :

*Toucher.*—In this form of inflammation and ulceration of the uterine neck, the toucher is a much more valuable means of diagnosis than in the former, or, at least, gives much more certain data. The finger very soon reaches the prolapsed cervix, which is only one, two, or three inches from the vulva, especially if the woman is standing, instead of four or five, as is naturally the case. The vagina is moistened by an abundant leucorrhoeal secretion, and is often hotter than usual. The increased size of the cervix is at once recognised, as also its resistance to pressure and great density. The os uteri is nearly always more or less open, so as to admit a small portion of the extremity of the finger, and the soft velvety sensation of the ulcerated surface is occasionally very evident, when the granulations are luxuriant or fungous.

Sometimes, if the disease is the result of difficult or instrumental labor, or of a miscarriage, the cervix is found deeply fissured, so as to present several lobes or lobules. When this occurs, even a practitioner who has had great experience in uterine disease, may be led to conclude that the affection is of a cancerous nature, unless he analyze very minutely the history and symptoms of the case ; the more so, as it is in such instances that the general symptoms are the most alarming. I have met with several cases of this kind, in which many of the symptoms of ulcerated uterine cancer were present, (cachectic cancerous tinge of the skin, extreme emaciation, abundant leucorrhœal discharge, often tinged with blood, occasional flooding, indurated, lobulated, and ulcerated cervix,) that it was with difficulty I could form a correct diagnosis. This may be done, however, if we attend to the history of the patient, and examine minutely the local state of the uterine organs. The origin of the disease may always be traced to difficult parturition. The fissures divide the cervx into lobules, but each lobule is itself smooth and round, however indurated it may be. The fissures radiate from the os. The vagina is perfectly free, at its union with the cervix, which is seldom the case in advanced ulcerated cancer. Moreover, in these extreme cases, the inflammation always extends deeply into the tissue of the uterus, the volume of which is increased.—By pushing back the posterior or anterior cul de sac of the vagina with the pulp of the index finger, it is not difficult to ascertain whether or not the induration of the cervix extends to the posterior or anterior plane of the uterus. In order to ascertain whether the volume of the uterus itself is increased, one or two fingers of the right hand must be introduced into the vagina, the pulp of the fingers directed towards the pubis of the patient. The fingers being then placed underneath the cervix, and the posterior vaginal cul de sac pused back, it is very possible to raise the uterus by them. If the left hand is placed at the same time on the hypogastic region, immediately above the pubis, and the patient is told to relax the abdominal parietes, the abdomen may be depressed over the uterus, so as for the latter organ to be distinctly felt between the finger in the vagina, and the hand over the pubis. Its volume may thus be very accurately appreciated. The ovaries, lateral ligaments, indeed, the entire pelvic cavity, may be explored in this way with the greatest ease. It is, however, absolutely necessary, to accomplish this exploration satisfactorily, that the examination should be made whilst the patient is lying on her back. The two hands could not be used freely if the patient were placed on her side, as is usnally the case in thiscountry.

*Speculum.*—The speculum reveals still more plainly the state of things. When the cervix has become the seat of inflammatory induration, whether caused or not by the ulceration, the existence of the induration tends to keep up the ulceration, and will invariably reproduce it if the latter be cured whilst it is suffered to remain. As I have stated, the extent and degree of intensity of the ulceration generally coincide with those of the engorgement. The ulceration itself is susceptible of presenting all the modifications which suppurating surfaces are capable of offering under different circumstances in other parts of thebody, from the minute granulations of a slight abrasion, to the livid fungous vegetations of an unhealthy sore. The distinction into herpetic, scorbutic, scrofulous, cancerous ulcerations, &c., which some authors(M. Duparcque and others) have proposed, has no practical utility whatever, and need scarcely arrest our attention for a moment. I shall therefore merely mention that tubercular ulceration is the ulceration which follows the softeningand rupture of tubercles situated in the uterine cervix. The softening of the tubercle occasions a little inflammation around it in the tissue of the neck. For sometime after it has emptied itself, the orifice of the tubercular cavity remains the seat of a circumscribed ulceration, and the cavity itself constitutes a little abscess, which, as is usually the case with a scrofulous abscess, is rather tardy in filling up. Tubercles are very rarely met with in this situation. M. Lisfranc has only seen four or five instances during the entire course of his immense practice. The diagnosis is founded on the presence of tubercles in other parts of the economy, and in that existence of the small circumscribed abscess of the cervix, containing caseous tubercular matter, which may be pressed out with the edge of the speculum.

In women who are pregnant, inflammation and ulceration of the cervix gives rise to the same symtoms as in those who are not. The existence of the disease is, however, seldom suspected ; the leucorrhœal dischage, the lumbar and hypogastric pains, and other abnormal sensations being nearly always attributed to the pregnancy. On examination with the speculum, the ulcerated surface is generally found to be covered with large, livid, fungous granulations, which bleed with the greatest ease. The portion of the cervix that is not ulcerated also presents a livid hue, which, it must be recollected, is natural to pregnant women. The engorgement is always considerable, its degree, however, depending, says M. Costilhes, *on the more or less advanced stage of the pregnancy*, an opinion which coincides entirely with the results furnished by my own experience. The engorgement is sometimes so great, and the ulcerated surface is covered with such unhealthy-looking granulations and vegetations, that M. Boys de Loury has repeatedly seen the disease mistaken for cancer.

With reference to the causes of diseases of the cervix the author remarks as follows :

CAUSES.—Out of twenty cases of non-venereal inflammation and ulceration of the cervix which we meet with in practice, seventeen may be distinctly traced to abortion or to labor, two will recognise other causes, and occur in women who have borne children, whilst only one will be found in females who have never conceived. I do not give this statement as the result of statistical researches, but as the impression left on my mind by the examination of a very large number of cases.

The rapid and extensive dilitation of the cervix during parturition is supposed to occasion the disease in a large portion of cases. This explanation, which is certainly plausible, we give in the author's words:

Let us see what takes place in the cavity of the *neck* of the uterus. Here we have a mucous membrane, the structure of which is, naturally, more perfect than that of the one lining the uterine cavity, and which, instead of being dissociated and apparently destroyed by the gradual enlargement of the uterus, becomes more vascular and perfect as pregnancy advances, and as the general organic vitality of the uterus increases. That such is really the case there can be no doubt, as dilatation of the os uteri only commences in primiparous women towards the end of the sixth month, and even in those who have born children, not until the end of the fifth. Moreover, the dilatation of the os uteri is very slight, until parturition actually commences, scarcely admitting the end of the index finger ; and is, most certainly, not calculated to interfere with the integrity of the mucous membrane which lines its cavity. As soon however, as the pains which precede and accompany the expulsion of the fœtus commence, the dialation of the os uteri progresses rapidly, and frequently in the course of a few hours it is carried to such an extent as to admit of the passage of the fœtus. Now it appears to me a necessary consequence of this rapid dilation of a canal lined by a mucous membrane in a state of integrity, that it must inevitably in many, if not in all cases, be accompanied by erosion, laceration, and contusion of the membrane. In the majority of women, no doubt, these lesions disappear promptly, cicatrization taking place with the greatest ease, under the influence of the retraction of the tissues of the neck, and of the reparative phlegmasia which sets up, after delivery, in the cervix, as well as in the body of the uterus. But if the physiological inflammation of the uterus which follows parturition should prolong its duration, and assume a pathological character ; if remnants of the placenta or of the membranes left in the uterine cavity give rise, by their decomposition, to an irritating fœtid discharge, it is easy to understand that the lesions of the mucous membrane, instead of healing, will almost inevitably become the seat of inflammation and of subsequent ulceration.

The chapters on syphilitic and cancerous ulcerations we pass by, not that they are unimportant, but they are of less practical moment, the former on account of their extreme infrequency, and the latter because of their obvious and irremediable character; and in endeavoring to give the *gist* of the other portions of the treatise, we shall occupy as much space as we can consistently appropriate to this subject in the present number. It remains to give an analysis of the methods of treatment recommended in affections of the cervix occuring in those who have borne children.

It is not to be forgotten that inflammation, &c., of the neck, especially when directly attributed to miscarriages, or difficnlt labor, may be connected with an inflamed condition of the body of the uterus, or metritis. Under these circumstances there is always more or less febrile reaction, which is rarely present when the cervix alone is inflamed. When this complication exists, general blood-letting, and other antiphlogistic measures, are called for; but these measures are seldom requisite when the inflammation is confined to the cervix.

In many cases the treatment already mentioned as adapted to inflammation and ulceration of the neck, without induration, will suffice in cases in which the latter lesion has ensued. This might rationally be supposed if the author's views respecting the dependence of the induration upon previous ulceration be adopted. *Sublata causa tollitur effectus.* When, however, the hypertrophy has been of long standing, perhaps having existed for years, the tissues have become too deeply modified to undergo restorement, even provided the inflammation and ulceration are cured. The patient will still remain liable to prolapsus, anteversion, and other distressing symptoms, as well as to relapses of inflammatory and ulcerated conditions of the part.

The treatment of inflammation and ulceration when complicated with induration, should be conjoined with more complete rest than when this complication does not exist. It will be better if the patient can be prevailed upon to keep her bed for a few weeks. If much hypogastic pains are present linseed poultices will afford great relief. Tepid, or cold hip baths twice a day, will be useful. If after pursuing this plan for a few weeks the induration be not found to subside, Dr. B. recommends the application of leeches to the uterine neck. The following is his description of the method of applying them:

After introducing an ordinary conical metal* speculum, wipe off the mucus which covers the surface of the cervix with a little lint or sponge, and then place the leeches in the interior of the instrument. Over the external orifice of the speculum, spread a piece of linen, which depress with the finger into the speculum. In the concavity thus formed, place some lint or cotton, and then, with the forceps, push the whole towards the uterine neck. The li nen carries the leeches before it, and presses them against the os uteri.—On pulling out the linen and the lint, with which the speculum was plugged, in the course of about ten minutes it will nearly always be found that all the leeches have taken. They generally fill well in this situation, and the flow of blood is often considerable after they have fallen.

Concerning the efficacy of leeches Dr. B. holds the following language:

The beneficial influence of leeches when applied to the cervix, like that of all other therapeutical agents used in the treatment of uterine disease, has been strenuously denied. I have, however, no hesitation in stating, that no other remedy with which I am acquainted posseses the same efficacy in preventing deep-seated inflammation of the cervix from passing into a state of chronic induration, a state incurable except by the most energetic means—which most practitioners are afraid to resort to. During three years that I was the pupil or house-physician, of M. Gendrin, at La Pitie, I either applied leeches to the cervix myself, or had them applied by my dressers, several times a week, to different women ; so that I have certainly had a good opportunity of forming an opinion as to their value. In private practice, I have since derived the same benefit

*Glass specula should never be used. They may break in the vagina, however strong.

from their use that I did on hospital patients. Six, eight, ten or twelve leeches may be applied at once, according to the effect wished to be produced, and they should be re-applied several times, at intervals of five, six, eight or ten days, when necessary, until the desised effect is produced. The leech punctures always heal readily. Their bite is not felt by the patient, unless they fix on the vagina, which they cannot do if the speculum is properly introduced. This instrument must be held by the patient or the nurse, while the leeches are on. They generally fall off but it is sometimes necessary to bring them away, after they have filled.

Dr. B. objects *in toto* to pessaries. In this we believe he is sustained by the majorify of the most judicious practitioners of the present day. Occurring as prolapsus does in the larger proportion of cases as a consequence of a diseased condition of the cervix, if we were to devise means to perpetuate this diseased condition, we could not do so more effectually than by introducing into the vagina a foreign body to be a constant source of irritation to the affected part. How this instrument conld have been in use so long, indiscriminately, in cases of prolapsus, when its effects are in so many cases so palpably injurious is surprising, and goes to illustrate the fact that the evil consequences of a remedy will not always open our eyes to its true character, while the the theoretical considerations upon which it is based continue in vogue.

But there will be some cases in which the treatment already laid down will prove ineffectual. The inflammation may be subdued, the ulcerations may heal, but the induration remains. What then is to be done? The answer to this question involves measures which will probably be to many of our readers novel, and which we suspect have been as yet, certainly in private practice, adopted in this country but to a very limited extent. These measures consist in deeply cauterizing the cervix either with the Vienna paste (quick lime and potassa fusa) or with the actual cautery. The object is to form an eschar; "the result of which is," to quote the author's words," that not only is the hypertrophy diminished by the extent of the eschar which separates, but the healthy inflammation set up in the chronically indurated tissues gradually melts them, as it were ; so that often, on its subsiding, the hypertrophied cervix has regained its natural size. When this result is not obtained by the first cauterization a a second or third seldom fails to reduce the uterine neck to its normal dimension. With the disappearance of the hypertrophy also disappear the symptoms which it occasioned; the uterus returns of itself to the position which it naturally occupies in the pelvis, and the cure is really accomplished.

The following is the method of applying the Vienna paste pursued by M. Gendrin, and adopted by ths author."

When applied to the uterine cervix, a large and conical speculum must first be introduced, and the engorged cervix made to enter its orifice; or should the cervix be too voluminous, the speculum must be firmly pressed on the part which it is intended to cauterize, great care being taken not to enclose between the rim of the speculum and the cervix a fold of the vagina. About as much of thepaste-as would cover a four-penny piece, a line in thickness, must be placed on a triangular piece of diachylon plaster, one end of which is inserted lightly into the cleft extremity of a small stick. The caustic paste is then carriod, by means of the stick, to the cervix, and applied to the centre of the part comprised by the orifice of the speculum. With the long forceps cotton is placed carefully all round the spot on which the caustic is applied, so as to completely protect the neighboring parts; the stick having been withdrawn, the speculum is two-thirds filled with cotton or lint, which is firmly pressed against the uterine neck. The speculum is then extracted, the cotton which fills it being forcibly pushed back in the vagina with the forceps, as it is pulled away, so that the vagina remains thoroughly plugged. If all this is carefully done, it is impossible for the caustic to fuse, and to injure the parietes of the vagina. In about fifteen or twenty minutes, the cotton or lint must be gradually withdrawn by means of a bivalve speculum, and an eschar of the size of a shilling, or rather larger, will be found where the caustic was applied. The vagina should then be washed out with a little tepid water, complete rest in bed enjoined, and emollient injections employed until the separation of the eschar, which takes place from the sixth, to the eighth or tenth day.

The use of the actual cautery to accomplish the same end is peculiar to M. Jobert. The operation as practiced by this surgeon is thus described:

He uses an ivory conical speculum, in order to protect the neighboring parts from the heat, ivory being a bad conductor of caloric. He then extinguishes on the part of the cervix which he wishes to cauterize, one, two, or three, olive-shaped cauteries, heated to whiteness, according to the depth to which he wishes to destroy the tissue of the cervix. A deep eschar is thus formed, as by cauterization with the Vienna paste. But little pain is experienced by the patient, and the eschar falls also from the sixth to the tenth day.

Its elimination is likewise accompanied by considerable inflammatory re-action in the indurated cervix, which, generally speaking, rapidly diminishes, or melts under the influence of the revival inflammatory process.

Sometimes the effect of one cauterization suffices to bring the uterine neck to its natural size, sometimes two or more are necessary.

The general effects of the application of the potential and actual cautery are represented to be but slight. Trifling hysterical symptoms are sometimes produced, which the author attributes to mental rather than physical causes. The inflammation produced by the application may extend to the uterus, but this is extremely rare. The author states that during the three years passed with M. Gendrin, at La Pitie, one or two patients were cauterized in this way (i. e. with the Vienna paste) every fortnight, and he does not recollect ever having witnessed any serious results. The suspicion that unhealthy ulcerations would be likely to follow the separation of the eschar is totally unfounded; nor is there the least danger of occlusion of the os uteri. The constant secretion of mucus issuing from the uterine cavity keeps this orifice open.

Finally we quote the author's concluding remarks on this subject:

To sum up : I firmly believe that chronic induration and hypertrophy of the cervix uteri, the result of inflamation and ulceration, will often be found incurable by any other means than excision or deep cauterization. Excision ought, I believe, to be be excluded, owing to the severe hæmorrhage which follows, and the danger which consequently attends it. Deep cauterization being resorted to, I prefer, in most cases, the Vienna paste to the actual cautery, but merely because it alarms the patient less, and has the appearance of a formidable operation. I have had such extensive experience of both agents in the Paris hospitals, and I think myself fully warranted in stating, that in the hands of careful and intelligent practitioners there is no more danger in resorting to deep cauterization of the cervix than in performing any other of the minor operations of surgery. I have also seen so many miserable women, who have suffered for years under engorgement of the neck of the uterus, (some of whom had all along been under treatment,) relieved and cured by deep cauterization alone, that I have no hesitation in recommending its adoption to the attention of my professional brethren.

It must not, however, be forgotten, that cauterization of the cervix, as above decribed, is *an operation*, and like *all* operations, surrounded with dangers ; that, consequently, it must neither be lightly undertaken or lightly carried through.

This article is already so extended that we will not lengthen it by comments on the foregoing plan of treatment. We will simply say that while we do not discredit the evidence of its safety, or propriety in some instances, we cannot but think that the cases are few in which it is called for. We trust we are correct in this surmise for we apprehend that most practitioners, like ourselves, will feel great reluctance to resort to the actual or potential cautery in private practice, except as a dernier resort, in some cases of peculiar severity.

Our sense of the interest and practical value of Dr. Bennet's work is sufficiently declared in our endeavor to present a digest of the most important portions of its contents. Those of our readers who may be led by the foregoing analysis to consult the work, will find, in addition to a more full enumeration of the views which have been presented in this article, a collection of cases illustrating the success of the methods of treatment which are recommended.

